# ROS-mediated membrane damage and antioxidant imbalance drive apple flesh browning during cold storage

**DOI:** 10.3389/fpls.2025.1718635

**Published:** 2025-12-01

**Authors:** Jihan Wang, Fujun Li, Bingru Li, Ling Li, Jing Shang, Xiaodong Fu, Xiuming Zhao, Xiaoan Li, Xinhua Zhang, Zienab F. R. Ahmed

**Affiliations:** 1 College of Agricultural Engineering and Food Science, Shandong University of Technology, Zibo, Shandong, China; 2 College of Food and Biological Engineering, Beijing Vocational College of Agriculture, Beijing, China; 3 Integrative Agriculture Department, College of Agriculture and Veterinary Medicine, United Arab Emirates University, Al Ain, United Arab Emirates

**Keywords:** apple flesh browning, cold storage, ROS metabolism, antioxidant imbalance, compartmentalization damage

## Abstract

**Introduction:**

Flesh browning is a major postharvest disorder in apples during cold storage, yet its physiological basis remains unclear. This study examined reactive oxygen species (ROS) metabolism and antioxidant systems in flesh-browning (FB) and normal (FN) tissues of browning apples, compared with healthy fruit (Control).

**Methods:**

‘Fuji’ apples were stored at −1-0°C for 120 days. Flesh color, membrane damage indicators, phenolic metabolism, antioxidant contents (AsA, GSH), ROS levels (H_2_O_2_, O_2_^•-^), and antioxidant enzyme activities (SOD, CAT, APX) were analyzed, together with relevant gene expression.

**Results:**

FB tissues showed the highest PPO and POD activities, enhanced membrane damage (higher EC, MDA, LOX), and markedly elevated ROS. Total antioxidant capacity decreased by ~25% in FB, accompanied by significant reductions in AsA and GSH and lower CAT/APX activities. Although phenylpropanoid-related enzymes and genes were upregulated, the resulting phenolics and flavonoids were insufficient for ROS mitigation. Strong correlations were found between browning intensity (a*), ROS accumulation, membrane damage, and antioxidant depletion.

**Discussion:**

Prolonged cold storage induces severe ROS accumulation and membrane disruption in FB tissues. Simultaneous reductions in non-enzymatic antioxidants (AsA/GSH) and antioxidant enzymes (CAT/APX) create a self-reinforcing imbalance in ROS metabolism, ultimately triggering enzymatic browning.

## 1 Introduction

Apple (*Malus domestica* Borkh) is a cultivated crop widely grown around the world, favored by consumers for its rich nutritional value and sweet-tart taste. As of 2023, global apple production has exceeded 97.34 million tons (Morariu et al., 2025). To ensure year-round availability and reduce post-harvest losses, a substantial portion of harvested apples are stored until they are sold. However, a persistent challenge in apple storage is the development of flesh browning (FB), a physiological disorder that compromises fruit quality and marketability (Gapper et al., 2023).

Recent studies have reported and reviewed different manifestations of apple flesh browning under storage conditions, typically linked to suboptimal temperature, gas composition, and storage duration (Sidhu et al., 2023). Damage to the cell membrane integrity together with loss of intracellular compartmentation are considered primary drivers of flesh browning, as these facilitate enzymatic reactions between polyphenol oxidase/peroxidase (PPO/POD) and phenolic compounds, ultimately leading to fruit and vegetable browning (Kuddus, 2018). Phenolic compounds, as important secondary metabolites in apples, play a dual role in flesh browning. On one hand, phenolics such as chlorogenic acid and catechin serve prime substrates for enzymatic oxidation, yielding brown pigments under the catalysis of PPO and POD (Vámos-Vigyázó and Haard, 1981). Alternatively, phenolic compounds function as antioxidants through the donation of hydrogen atoms or electrons, effectively neutralizing free radicals, and by enhancing antioxidant enzyme activities to suppress reactive oxygen species (ROS) generation, thereby mitigating ROS-mediated oxidative damage and membrane lipid peroxidation (Cosme et al., 2020).

In addition, other non-enzymatic antioxidants and ROS-scavenging enzymes also play crucial roles in the process of flesh browning. For instance, ascorbic acid (AsA), which functions as an efficient inhibitor of phenolases, exhibits strong anti-browning capabilities (Liu et al., 2021; Ali et al., 2015). Reduced glutathione (GSH) can react with quinones to produce colorless substances, thereby inhibiting the occurrence of browning (Vámos-Vigyázó and Haard, 1981; Ali et al., 2025). Superoxide dismutase (SOD), one of the key ROS-detoxifying enzymes, converts superoxide anion radicals (O_2_^•^−^^) into hydrogen peroxide (H_2_O_2_). The resulting H_2_O_2_ is further eliminated through its breakdown into water by catalase (CAT) and ascorbate peroxidase (APX), thereby protecting plant tissues from oxidative stress (Zhu et al., 2022; Lee et al., 2019).

This study focused on checking out the variations in the metabolic profiles of ROS, together with both enzymatic and non-enzymatic antioxidants among flesh-browning (FB) and normal (FN) tissues of browning apples, as well as healthy apple flesh (Control) tissues, aiming to elucidate their potential contributions to the onset of flesh browning during apple cold storage.

## 2 Materials and methods

### 2.1 Materials

‘Fuji’ apples at commercial maturity were harvested on October 15, 2022, from an orchard in Yantai (37°08′27″N, 121°01′02″E). Three hundred undamaged fruits were selected and maintained in a local commercial cold storage (-1~0 °C, 90% RH). After 120 days, all the apples were collected and transported to Shandong University of Technology.

**Figure 1:**
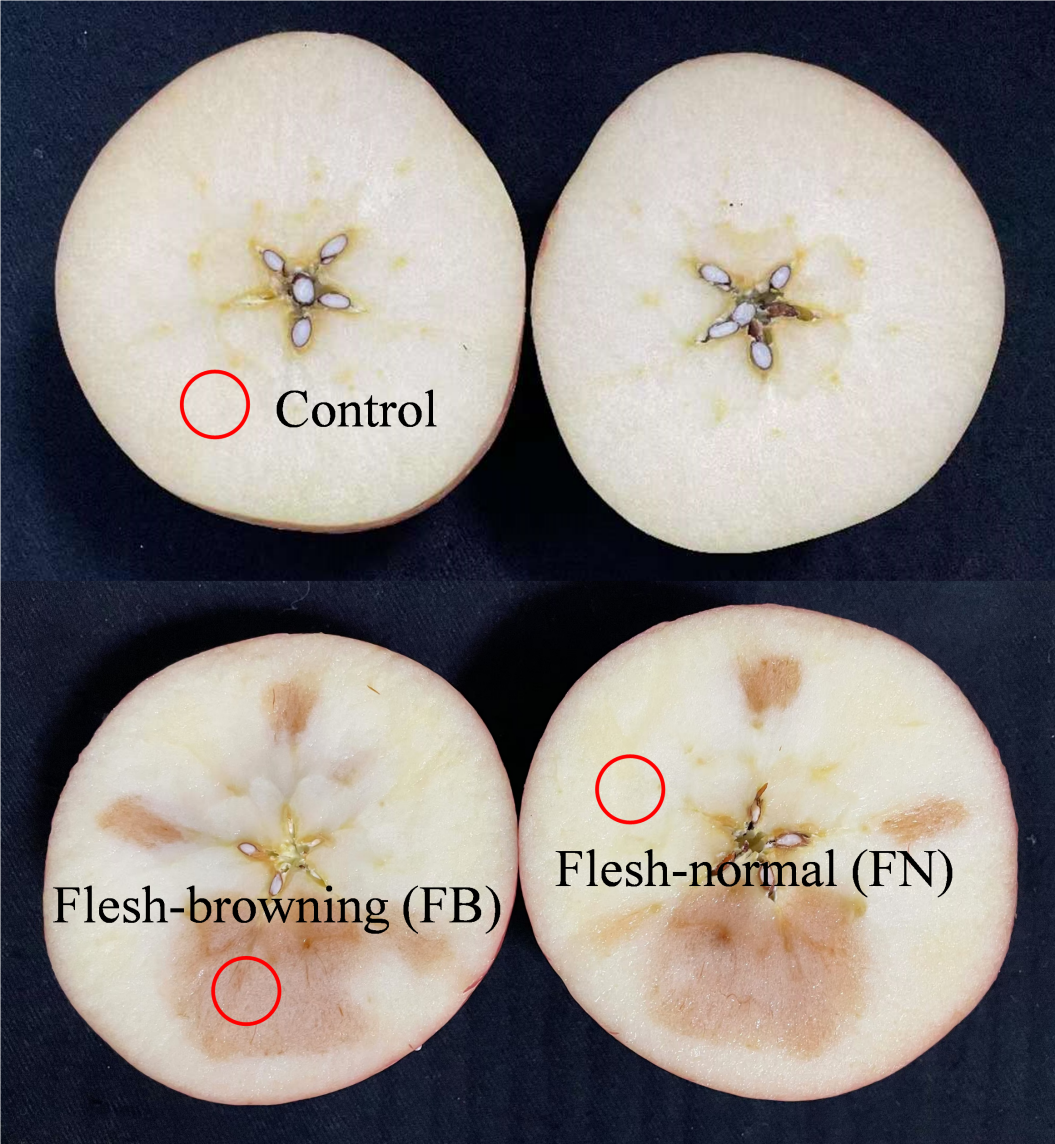
Control, flesh-normal (FN), and flesh-browning (FB) samples of ‘Fuji’ apple fruit after cold storage (-1~0°C, 90% RH) for 120 d.

All apples were cut along the equatorial section, and from these, ten healthy and ten flesh-browning apples were randomly selected for analysis. The FB and FN from the flesh-browning apples were used as experimental samples, while the healthy flesh (Control) tissues were taken from healthy apples (**Figure 1**). Fresh samples were allocated for the evaluation of fruit quality parameters such as color difference, flesh-firmness, electrical conductivity (EC), titratable acidity (TA), and soluble solids content (SSC). The remaining samples were rapidly frozen in liquid nitrogen and subsequently stored at -80°C.

### 2.2 a*, flesh firmness, SSC and TA

Following the procedure described by Wang et al. (2024), the a* value of apple flesh was assessed with a colorimeter (KONICA MINOLTA, CR-400, Japan). Flesh -firmness was evaluated with a texture analyzer (TMS-2000, Los Angeles, USA) fitted with a probe of 5 mm in diameter, descending at 3 mm s^-1^ with an 8 mm penetration depth. Results were expressed in g cm^-2^. Sugar content in the juice squeezed from flesh tissues was quantified using a handheld Refractometer (PAL-1, ATAGO, Japan). TA was determined through titration with 0.01 M NaOH to pH 8.1, employing phenolphthalein as the indicator, following Li et al. (2013). TA was calculated based on NaOH volume consumed. Both SSC and TA were expressed as percentages (%).

### 2.3 Activities of PPO and POD

Two grams of fruit tissue were homogenized in 5 mL of 50 mM sodium phosphate buffer (pH 7.0) containing 1 mM PEG, 4% PVPP, and 1% Triton X-100. The resulting supernatant was collected and used for enzyme assays. POD activity was determined by the guaiacol assay according to Luo et al. (2012), monitoring absorbance changes at 470 nm over 3 minutes. For PPO, enzyme solution (0.5 mL) was mixed with 50 mM phosphate buffer (2 mL, pH 6.8), then 0.1 M catechol (1 mL) was added. Absorbance change at 420 nm was recorded for three minutes (Zhang et al., 2017). One unit (U) of POD or PPO was defined as the amount of enzyme catalyzing the oxidation of 1 μmol of guaiacol or catechol per minute, respectively, and activities were expressed as U g^-1^.

### 2.4 Malondialdehyde content, EC, and lipoxygenase activity

The MDA content and EC were determined according to our previous method (Wang et al., 2023). LOX activity was assayed according to the protocol reported by Cao et al. (2007). Fruit tissue (5 g) was homogenized with 5 mL of 100 mM phosphate buffer (pH 6.8) containing 1% Triton-X100 and 4% PVPP, and the supernatant was collected after centrifugation. The reaction buffer (2.8 mL) for LOX was composed of 2.75 mL of 100 mM acetic acid–sodium acetate solution (pH 5.5) and 0.05 mL of 100 mM sodium linoleate. After incubating the reaction buffer at 30°C for 10 minutes, crude enzyme solution (0.2 mL) was added and mixed well. The variation in absorbance at 234 nm over a 3-minute period was monitored. One U of LOX activity was described as the quantity of enzyme required to produce a 0.01 increase in absorbance per minute. LOX activity was expressed as U kg^-1^.

### 2.5 Total antioxidant capacity

Frozen tissues (1.5 g) were ground with 5 mL distilled water, and the supernatant was obtained after centrifugation. TAC was assessed through the ferric reducing antioxidant power (FRAP) assay (Sethi et al., 2020), with FeSO_4_ (0–1 mM) used to establish the standard curve. For each sample, TAC was calculated as the FRAP value equivalent to the absorbance of 1 mM FeSO_4_.

### 2.6 Contents of total phenolics and total flavonoids

TP and TF contents were determined according to Li et al. (2023). Frozen tissues (2 g) were ground in a mortar with 5 mL methanol. After centrifugation, the supernatant was obtained, and its absorbance was recorded at 765 nm and 430 nm, respectively. Calibration curves were established using gallic acid (0–1 mM) for TP and rutin (0-0.2 mM) for TF, respectively. The results were expressed as g kg^-1^.

### 2.7 Analysis of phenylalanine metabolizing enzyme activities

Frozen tissues (2 g) were homogenized separately using three different Tris-HCl extraction buffers (5 mL): 100 mM (pH 8.8) for PAL extraction (Cheng and Breen, 1991), 50 mM (pH 8.9) for C4H extraction (Li et al., 2019), and 50 mM (pH 8.0) for 4CL extraction (Knobloch and Hahlbrock, 1975). The homogenates were centrifuged at 10,000 × g for 15 min at 4°C, and the resulting supernatant was used as a crude enzyme extract for subsequent activity determinations, following the procedure of Li et al. (2019). Enzyme activity was quantified by defining one U as the amount of enzyme that induces a 0.01 change in absorbance per minute, with results expressed as U kg^-1^.

### 2.8 Contents of AsA, dehydroascorbate, GSH and oxidized glutathione

Frozen tissues (2 g) were ground with 6% trichloroacetic acid, and the supernatant was obtained after centrifugation. AsA and total AsA in the flesh were quantified as described by Kampfenkel et al. (1995), using AsA standard solutions to establish a calibration curve (0-100 μg mL^−1^). DHA content was determined by the formula: DHA = total AsA - AsA.

Frozen tissue (2 g) was homogenized in 7% sulfosalicylic acid and centrifuged to obtain the extract. For total GSH, the reaction system was prepared according to the method described by Fu et al. (2023). After incubation at 27°C for 30 min, the absorbance of the mixture was measured at 412 nm. Determination of GSSG was conducted similarly, except that 2-vinylpyridine (0.1 mM) was included and the incubation extended to 1 h. Calibration curves were prepared using GSH standard solutions at concentrations of 0-100 μM, with and without 2-vinylpyridine treatment for GSSG and total GSH, respectively. The GSH content was determined by the formula: GSH = total GSH - GSSG.

Contents of AsA-GSH cycle related substances were expressed as μmol kg^-1^.

### 2.9 Detection of H_2_O_2_, and superoxide anion (O_2_^•-^)

For H_2_O_2_, 1.5 g of tissue was homogenized in acetone, and 1 mL of the extract was reacted with 0.2 mL of 5% (w/v) Ti(SO_4_)_2_ and 0.2 mL of concentrated ammonia to form a precipitate, which was washed with acetone and dissolved in 5 mL of 2 M sulfuric acid. The absorbance was measured at 415 nm (Wang et al., 2023). For O_2_^•-^, 1 g of tissue was homogenized in 5 mL of 50 mM phosphate buffer (pH 7.8). The extract (1 mL) was reacted with 1 mL of 1 mM hydroxylamine hydrochloride at 25°C for 1 h, followed by addition of 1 mL of 17 mM sulfanilic acid and 1 mL of 7 mM α-naphthylamine for 20 min at 25°C. The absorbance was measured at 530 nm (Wang et al., 2023).

H_2_O_2_ content was represented in mmol kg^-1^, while the production rate of O_2_^•-^ was expressed as mmol kg^-1^ min^-1^.

### 2.10 Analysis of antioxidant enzymes

Frozen tissues (1 g) were homogenized in 5 mL of phosphate buffer (50 mM). For SOD and CAT extraction, the buffer was adjusted to pH 7.8 and contained 10% (w/v) PVPP and 1 mM EDTA, while for APX determination, the buffer was set to pH 7.0. The homogenates were centrifuged at 10,000 × g for 10 min at 4°C, and the supernatant was used for enzyme activity analysis.

The activities of SOD, CAT, and APX were assayed as described by Li et al. (2022b). Specifically, each 1 mL SOD reaction system contained 50 mM phosphate buffer (pH 7.8), 13 mM methionine, 75 µM NBT, 0.05 mL enzyme extract, and 0.1 mM EDTA; CAT (1 mL) contained 50 mM phosphate buffer (pH 7.8), 20 mM H_2_O_2_, and 0.05 mL enzyme extract; APX (1 mL) contained 50 mM phosphate buffer (pH 7.0), 0.1 mM EDTA, 0.4 mM AsA, 0.1 mM H_2_O_2_, and 0.05 mL enzyme extract. Absorbance changes were recorded continuously for 15 min at 5-min intervals for SOD (560 nm) and for 3 min at 30-s intervals for CAT (240 nm) and APX (290 nm).

One U of SOD, CAT, and APX was defined as the amount of enzyme that causes a change in absorbance of per second. Enzyme activities were expressed as U kg^−1^.

### 2.11 Quantitative reverse transcription PCR analysis

Total RNA extraction, cDNA synthesis, and qRT-PCR were conducted based on our previously reported methods (Wang et al., 2024). Primer sequences for qRT-PCR are provided in **Supplementary Table S1**, and relative gene expression was determined using the 2^-ΔΔCT^ method.

### 2.12 Statistical analysis

Statistical analysis of the data was conducted using one-way ANOVA with Ducan’s multiple range tests in SPSS software (version 12.0). Data are presented as means ± standard errors based on three replicate, and statistical significance was defined at *p* < 0.05.

## 3 Results

### 3.1 a*, flesh firmness, SSC and TA

As presented in **Table 1**, the a* value for FB (10.87) was markedly elevated compared to those of FN (3.7) and Control (3.27), which indicates a pronounced degree of browning in FB. Flesh firmness was measured with a texture analyzer, showing significantly higher values in browning-fruits than in the Control, but independent of whether tissues were browned or not (**Table 1**). The SSC and TA serve as essential components for fruit metabolic processes. Among the three samples, the SSC and TA concentrations in FB were the lowest, showing reductions of 68.42% and 28.66% relative to the Control, respectively. In FN, the corresponding decreases were 10.53% and 13.62% compared with the Control (**Table 1**).

**Table 1:** a*, Flesh-firmness, and contents of SSC and TA in the browning or non-browning flesh of ‘Fuji’ apple fruit during cold storage.

Name	a*	Firmness-flesh (g cm^-2^)	TA (%)	SSC (%)
Control	3.27 ± 0.29^b*^	243.88 ± 5.39^b^	0.19 ± 0.00^a^	14.90 ± 0.14^a^
FN	3.7 ± 0.29^b^	265.84 ± 14.52a^b^	0.17 ± 0.00^b^	12.87 ± 0.45^b^
FB	10.87 ± 0.41^a^	292.42 ± 19.99^a^	0.06 ± 0.00^c^	10.63 ± 0.19^c^

^*^Different letters in the same line mean significant difference at *p* < 0.05.

### 3.2 Activities of PPO and POD

**Figure 2:**
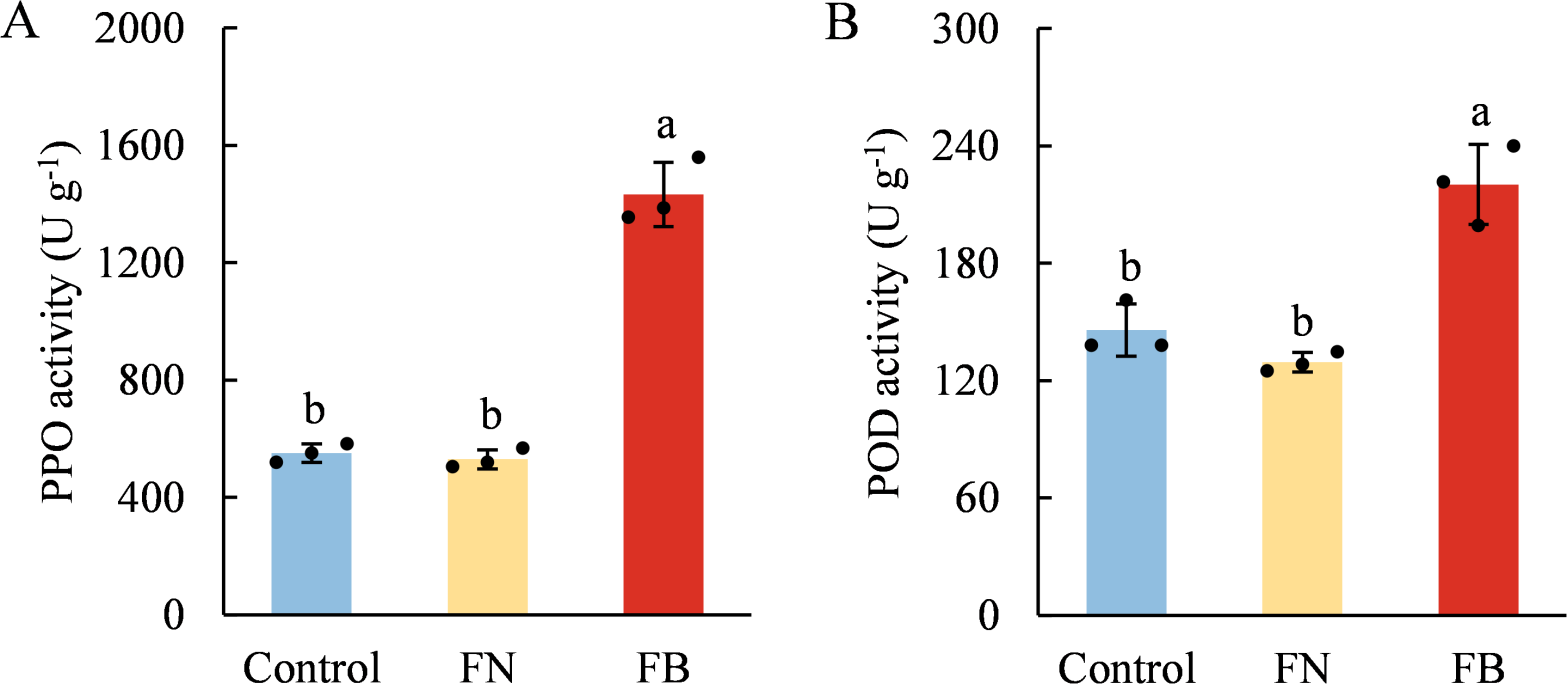
Enzyme activities of polyphenol oxidase (PPO) **(A)** and peroxidase (POD) **(B)** in browning or non-browning flesh of ‘Fuji’ apple fruit. Different lowercase letters indicate significant differences between data (*p* < 0.05). x-axis: represents different flesh samples.

PPO and POD demonstrated similar trends across the three fruit flesh tissues, both of which had the highest enzyme activity in FB. Specifically, PPO activity was 159.85% higher than Control and 170.08% higher than FN, while POD activity was 51.08% and 70.45% higher than Control and FN, respectively (**Figure 2**).

### 3.3 MDA content, EC, LOX activity, and TAC

**Figure 3:**
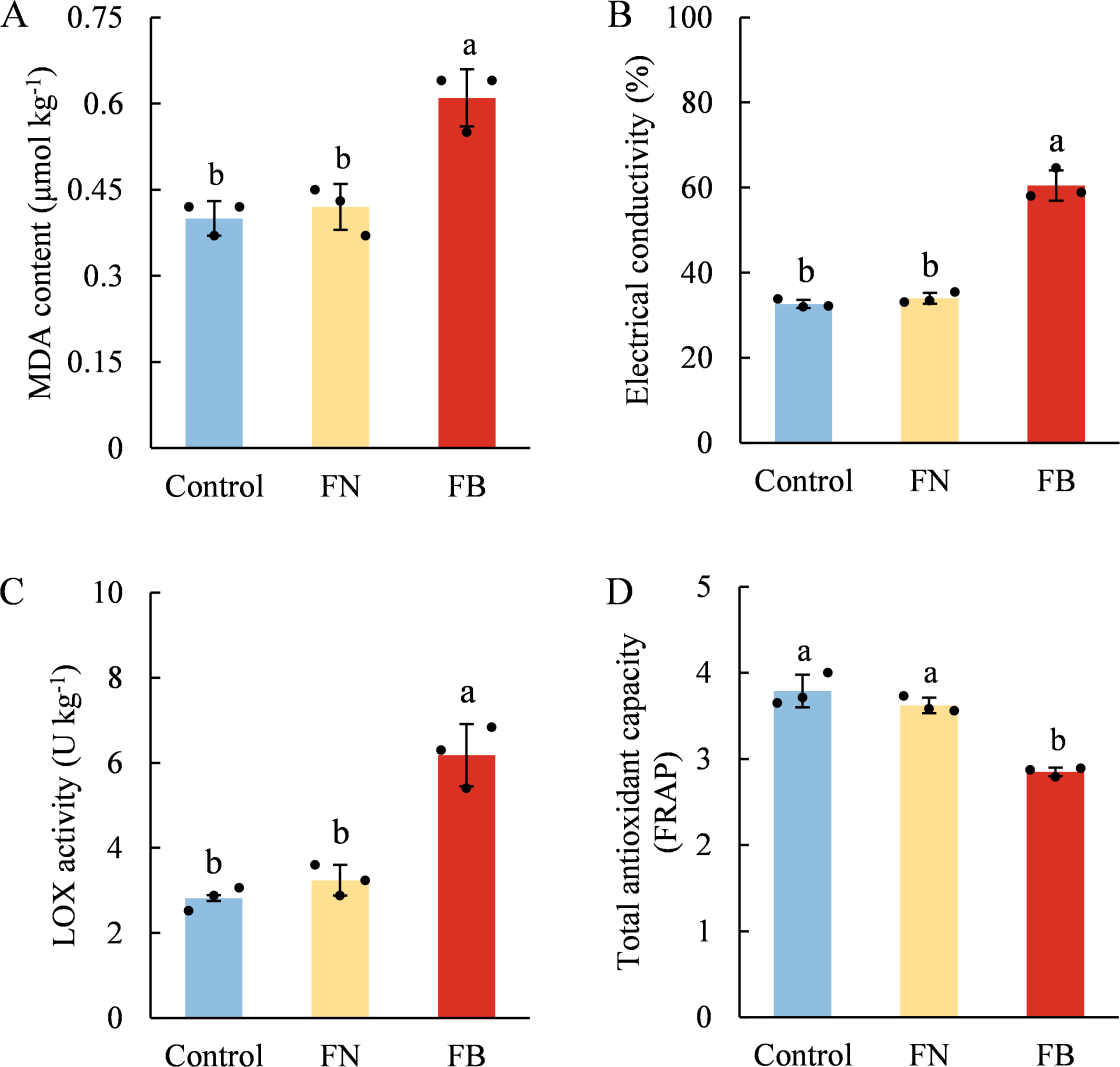
Malondialdehyde content (MDA) **(A)**, electrical conductivity (EC) **(B)**, lipoxygenase (LOX) activity **(C)**, and total antioxidant capacity (TAC) **(D)** in browning or non-browning flesh of ‘Fuji’ apple fruit. Different lowercase letters indicate significant differences between data (*p* < 0.05). x-axis: represents different flesh samples.

Compared with non-browning tissues, the EC, MDA content and LOX activity in FB were highest (**Figures 3A-C**). TAC levels in the Control and FN remained at relatively high levels, with no significant difference observed between them, while the TAC in FB exhibited a marked decrease, amounting to only 75.2% and 78.73% observed in the Control and FN, respectively (**Figure 3D**).

### 3.4 Contents of TP and TF

**Figure 4:**
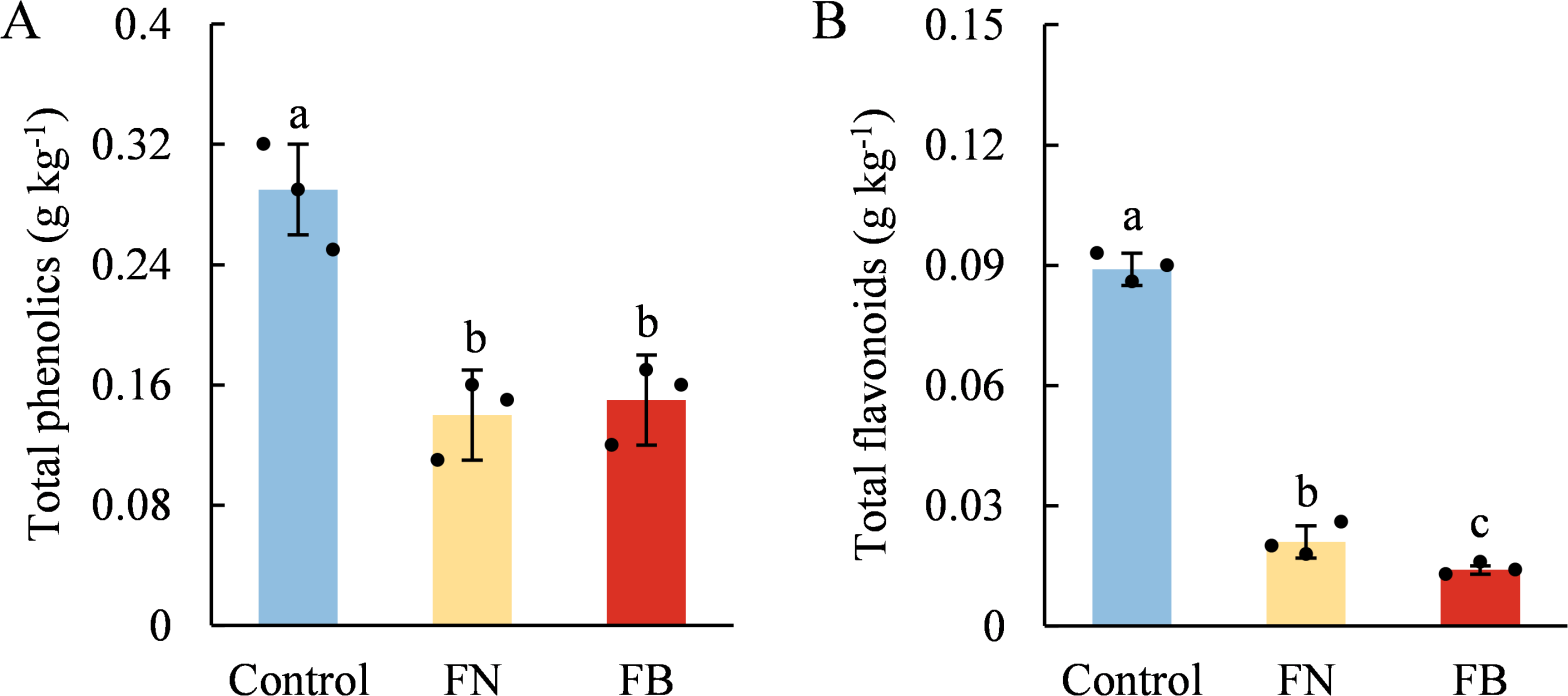
Contents of total phenols (TP) **(A)** and total flavonoids (TF) **(B)** in browning or non-browning flesh of ‘Fuji’ apple fruit. Different lowercase letters indicate significant differences between data (*p* < 0.05). x-axis: represents different flesh samples.

TP contents in the flesh tissues of browning-fruit were significantly lower than that in the flesh tissues of healthy fruit, while no significant difference was detected within browning-fruit (*p* > 0.05) (**Figure 4A**). TF contents showed significant differences among the three samples, with FB exhibiting the lowest level. TF levels in FB accounted for merely 16.13% of the Control and 68.47% of FN (**Figure 4B**).

### 3.5 Activities of PAL, C4H, and 4CL

**Figure 5:**
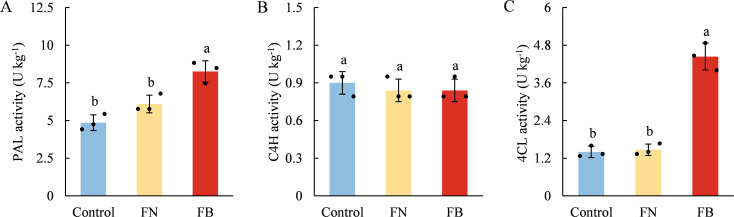
Enzyme activities of PAL **(A)**, C4H **(B)**, and 4CL **(C)** in browning or non-browning flesh of ‘Fuji’ apple fruit. PAL, phenylalanine ammonia-lyase; C4H, cinnamate 4-hydroxylase; 4CL, 4-coumarate, CoA ligase. Different lowercase letters indicate significant differences between data (*p* < 0.05). x-axis: represents different flesh samples.

The activities of PAL, C4H, and 4CL in the three samples were depicted in **Figure 5**. PAL and 4CL activities were significantly elevated in FB compared to those in non-browning tissues. FB, PAL activity exceeded that of the Control and FN by 69.96% and 35.41%, respectively, whereas 4CL activity was elevated by 217.14% and 202.04% relative to the Control and FN (**Figures 5A, C**). In contrast, C4H activity remained statistically unchanged across the three groups (*p* > 0.05) (**Figure 5B**).

### 3.6 Relative expression of genes involved in phenolic metabolism

**Figure 6:**
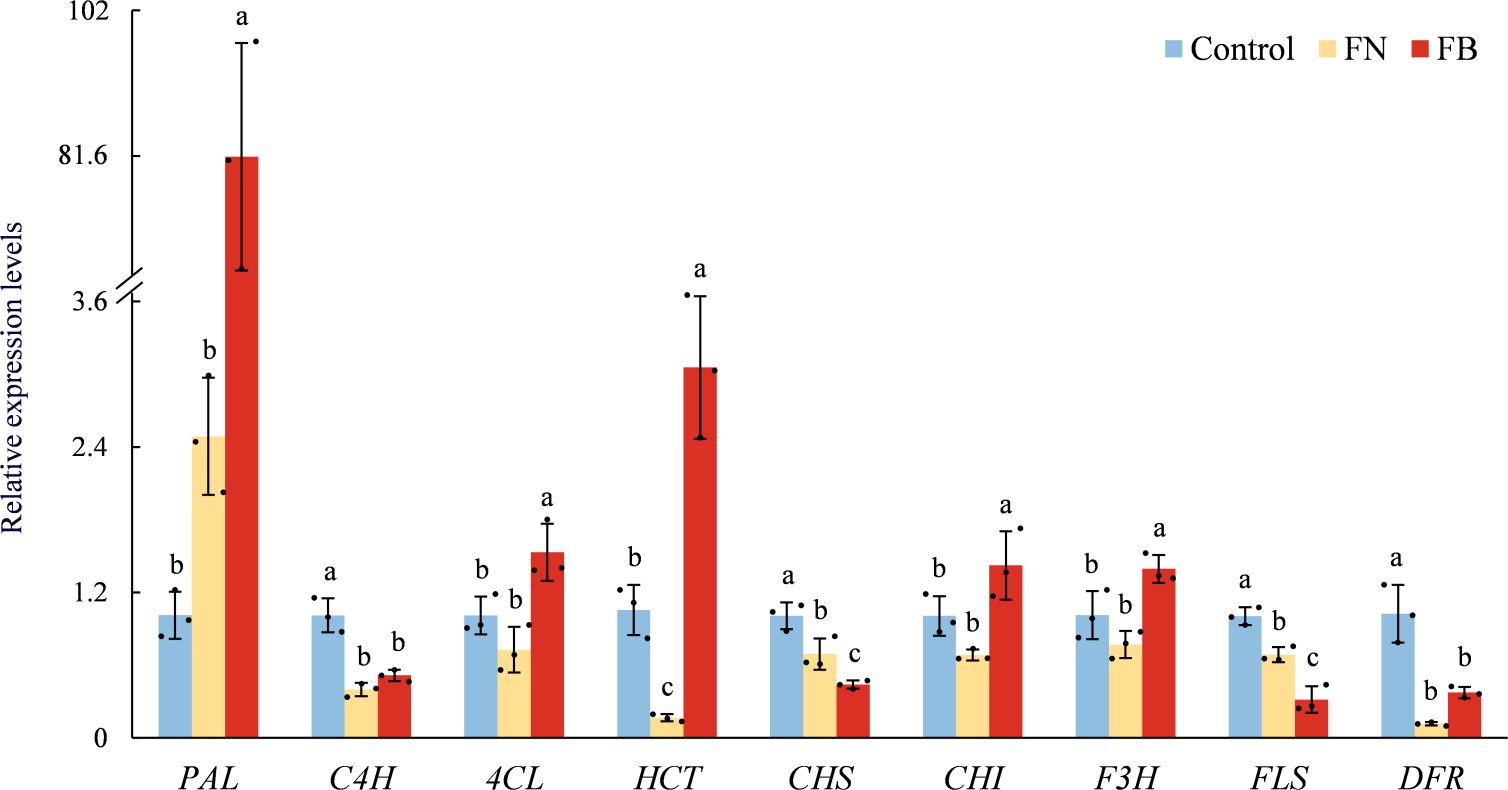
Relation expression of *PAL*, *C4H*, *4CL*, *HCT*, *CHS*, *CHI*, *F3H*, *FLS*, and *DFR* in browning or non-browning flesh of ‘Fuji’ apple fruit. PAL, phenylalanine ammonia-lyase; C4H, cinnamate 4-hydroxylase; 4CL, 4-coumarate:CoA ligase; HCT, hydroxycinnamoyl-CoA:shikimate hydroxycinnamoyl transferase; CHS, chalcone synthase; CHI, chalcone isomerase; F3H, flavanone 3-hydroxylase; FLS, flavonol synthase; DFR, dihydroflavonol 4-reductase. Different lowercase letters indicate significant differences between data (*p* < 0.05). x-axis: represents different gene names.

Relative expression of nine phenolic metabolism-related genes is shown in **Figure 6**. Overall, four genes (*PAL*, *C4H*, *4CL*, and *HCT*) related to phenolic acid metabolism were upregulated in FB, while the other five genes (*CHS*, *CHI*, *F3H*, *FLS*, and *DFR*) related to flavonoid metabolism showed exhibited varied expression profiles across the three sample groups. In FB, *PAL* expression was significantly elevated, by 7952.5% and 3176.05% relative to Control and FN, respectively. In addition, *4CL*, *HCT*, *C4H*, *CHI*, and *F3H* were also up-regulated to varying extents in FB, with the relative expression levels of *HCT* being 189.75% above the Control and 1745.27% greater than FN, respectively.

### 3.7 Contents of AsA, DHA, GSH and GSSG

As illustrated in **Table 2**, both the ratios of AsA to total AsA and GSH to total GSH exhibit the lowest levels in the FB. Notably, there were significant differences in AsA content among the three sample groups, with the FB showing the lowest content. DHA content in both Control and FN was slightly higher in comparison to FB, yet the differences among the groups were not significant (*p* > 0.05). Similarly, GSH and GSSG levels remained comparable between non-browning tissues (*p* > 0.05). By contrast, FB showed a marked reduction in GSH compared with non-browned tissues, whereas GSSG displayed the reverse pattern.

**Table 2:** AsA, DHA, GSH, and GSSG contents in the browning or non-browning flesh of ‘Fuji’ apple fruit during cold storage.

Name	Control	FN	FB
AsA (μmol kg^-1^)	181.90 ± 5.71^c^	164.87 ± 2.31^b^	148.13 ± 5.71^a^
DHA (μmol kg^-1^)	33.66 ± 1.90^a^	30.88 ± 1.10^a^	30.23 ± 1.85^a^
GSH (μmol kg^-1^)	31.48 ± 1.72^a^	31.30 ± 0.52^a^	23.52 ± 0.69^b^
GSSG (μmol kg^-1^)	2.59 ± 0.26^b^	2.96 ± 0.26^b^	4.44 ± 0.45^a^
AsA/(AsA+DHA)	0.86 ± 0.01^a^	0.84 ± 0.01^a^	0.80 ± 0.03^b^
GSH/(GSH+GSSG)	0.92 ± 0.01^a^	0.91 ± 0.01^a^	0.80 ± 0.01^b^

^*^Different letters in the same line mean significant difference at *p* < 0.05.

### 3.8 ROS metabolism system

**Figure 7:**
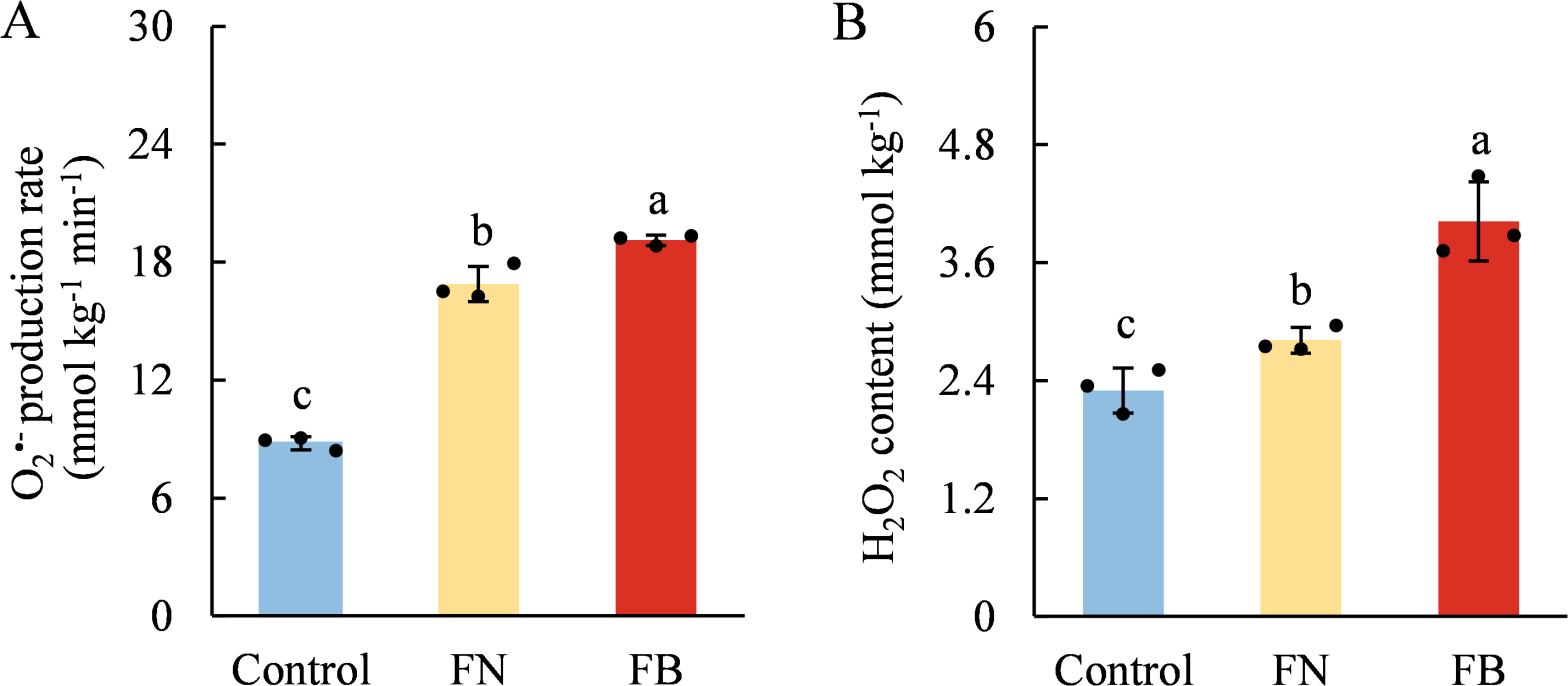
Schematic diagram of reactive oxygen species (ROS) metabolism and ascorbic acid (AsA)-reduced glutathione (GSH) cycle of ‘Fuji’ apple fruit during cold storage for 120 d. Heatmap based on metabolite levels in Control, FN, and FB tissues. ROS, reactive oxygen species; SOD, superoxide dismutase; CAT, catalase; APX, ascorbate peroxidase; MDHAR, monodehydroascorbate reductase; NADP^+^, nicotinamide adenine dinucleotide phosphate; NADPH, nicotinamide adenine dinucleotide phosphate; GR,glutathione reductase; GSSG, oxidized glutathione; DHAR, dehydroascorbate reductase; DHA, dehydroascorbate. Different lowercase letters indicate significant differences between data (*p* < 0.05). x-axis: represents different flesh samples.

**Figure 8:**
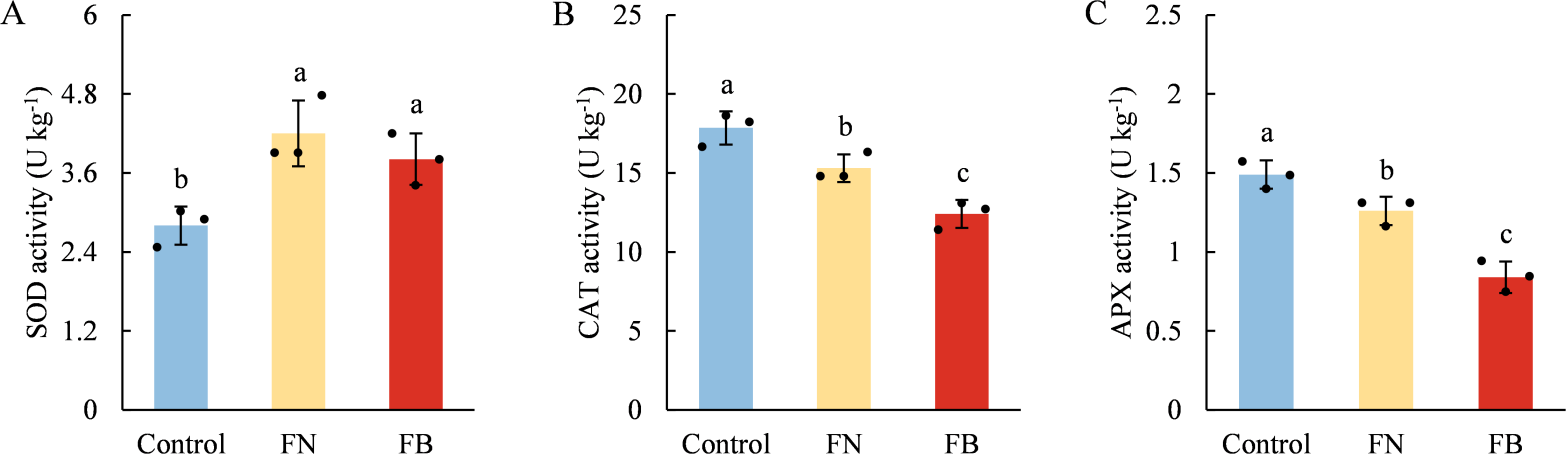
Pearson’s correlations analysis. Correlation heat map drawn based on the ROS, TAC, non-enzyme antioxidants and antioxidant enzyme levels, as well as a* in fruit flesh tissues. Each circle represents the correlations among all parameters with a color scale. 1-3 * represent significance *p* < 0.05, *p* < 0.01, and *p* < 0.001, respectively. SOD, superoxide dismutase; GSSG, oxidized glutathione; LOX, lipoxygenase; MDA, malondialdehyde; DHA, dehydroascorbate; TP, total phenolics; TF, total flavonoids; AsA, ascorbic acid; CAT, catalase; APX, ascorbate peroxidase; TAC, total antioxidant capacity; GSH, reduced glutathione. Different lowercase letters indicate significant differences between data (*p* < 0.05). x-axis: represents different flesh samples.

As shown in **Figure 7**, both H_2_O_2_ content and O_2_^•-^ production rate were significantly higher in FB than in non-browning flesh. Moreover, the flesh of browning-fruit exhibited higher SOD activity than the Control. In contrast, CAT and APX activities were lowest in FB and highest in the Control group (**Figure 8**).

### 3.9 ROS metabolism system

**Figure 9:**
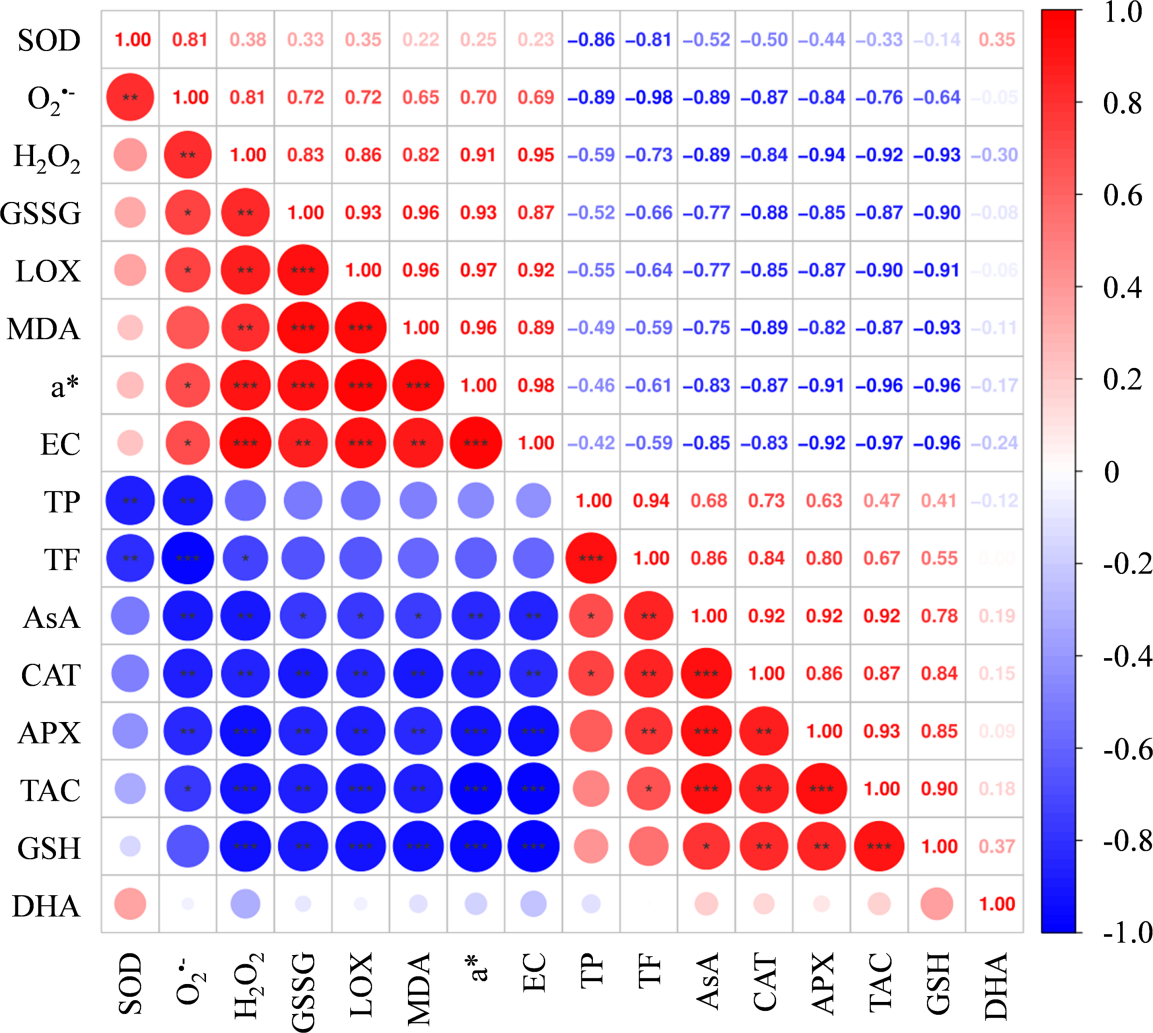
ROS-mediated membrane damage and antioxidant imbalance drive apple flesh browning during cold storage. The red and green arrows indicate an increase or decrease in substance content or enzyme activity, respectively. LOX, lipoxygenase; ROS, reactive oxygen species; PPO, polyphenol oxidase; POD, peroxidase; AsA, ascorbic acid; GSH, reduced glutathione; GSSG, oxidized glutathione; GR, glutathione reductase; DHAR, dehydroascorbate reductase; DHA, dehydroascorbic acid; MDHAR, monodehydroascorbate reductase; MDHA, monodehydroascorbic acid; APX, ascorbate peroxidase; CAT, catalase; SOD, superoxide dismutase; PAL, phenylalanine ammonia-lyase; C4H, cinnamate 4-hydroxylase; 4CL, 4-coumarate:CoA ligase. *, **, and ***indicate significance at *p* < 0.05, 0.01, and 0.001, respectively.

Correlation analysis among a*, ROS, TAC, as well as several antioxidants and antioxidant enzymes in the three groups was shown in **Figure 9**. The a* value exhibited significant positive correlations with the levels of O_2_^•-^, H_2_O_2_, GSSG, LOX, EC and MDA, while significant negative correlations with the levels of TAC, AsA, GSH, CAT and APX. Unexpectedly, no significant correlations were observed between a* and the contents of TP and TF (*p* > 0.05).

Antioxidants such as TF, AsA, and GSH, along with activities CAT and APX, made significant contributions to the TAC in the flesh tissues. Except for GSH, all demonstrated significant negative correlations with both O_2_^•-^ and H_2_O_2_ simultaneously. Among them, the correlations between TAC in the flesh tissues and O_2_^•-^ and H_2_O_2_ were -0.762 and -0.916, respectively (**Figure 9**).

## 4 Discussion

Apple flesh browning is a frequent postharvest disorder, typically linked to fruit senescence as well as storage conditions such as elevated CO_2_ or chilling stress (Sidhu et al., 2023; Gapper et al., 2023). Recently, a browning disorder similar to the soggy breakdown (SB) phenotype in ‘Honeycrisp’ has been increasingly observed in ‘Fuji’ apples during postharvest cold storage, leading to substantial deterioration of fruit quality (**Table 1**) and marketability. However, the underlying causes and pathogenesis of this browning disorder remain largely unresolved.

At present, the prevailing view suggests that disruption of cell membrane compartmentalization constitutes the major mechanism underlying enzymatic browning in fruit and vegetables (Kuddus, 2018). It is widely known that PPO is typically bound to thylakoid membranes in a latent (inactive) form, while phenolic compounds are located within vacuoles, separated by intact cellular membrane (Yoruk and Marshall, 2003; Glagoleva et al., 2020). Membrane permeability alterations or tissue integrity loss during senescence (Zhang et al., 2025), mechanical injury, or stress (Tang et al., 2020; Li et al., 2022b) can release and activate PPO, facilitating its interaction with phenolic compounds and thereby inducing browning. In addition, POD catalyze the oxidation of phenolic substrates, including ortho-diphenols and anthocyanins, in the presence of H_2_O_2_, thereby leading to melanin formation (Sultan et al., 2025). Earlier investigations have demonstrated that enhanced POD activity during cold storage contributes synergistically to peel or flesh browning in fruit and vegetables, including peaches (Badrunnesa et al., 2025), bananas (Wang et al., 2021), and bamboo shoots (Luo et al., 2012). Consistent with the above findings, our results showed markedly elevated PPO and POD activities in FB tissues, while no significant difference was detected between the non-browning tissues (**Figure 2**). Therefore, these data suggest that enzymatic browning triggered by PPO and POD may be a direct factor contributing to the flesh browning of apples, potentially associated with cell membrane damage induced by senescence or stress during cold storage conditions.

Our previous targeted metabolomic and transcriptomic analyses indicated that the damage of cell membrane structure in FB tissues might be associated with imbalanced ROS metabolism (Wang et al., 2023). Similar phenomena have been reported in pears (Li et al., 2022a), plums (Sogvar et al., 2020), water chestnuts (Zhu et al., 2022), and *Agaricus bisporus* (Dokhanieh and Aghdam, 2016), where excessive ROS production induced by low temperature, high CO_2_ levels, mechanical injury, or senescence accelerated the disruption of cellular membrane compartmentalization, ultimately leading to flesh browning. In this study, LOX activity in FB tissues was significantly induced, accompanied by increased EC, MDA and H_2_O_2_ contents, as well as elevated O_2_^•-^ production rates (**Figures 3A-C** and **Figure 7**). LOX, a copper-dependent enzyme, catalyzes the oxidation of unsaturated fatty acids, generating hydroperoxide and free radicals (He et al., 2020). MDA is a lipid peroxidation product commonly used to assess membrane lipid peroxidation and, together with EC, indicates cellular membrane injury (Wang et al., 2023). Earlier research has shown that LOX activity in fruit and vegetables is markedly affected by low-temperature stress (Hu et al., 2022; Mao et al., 2007; Feys et al., 1980) as well as extended storage time (Zhou et al., 2020; Mirshekari et al., 2019). For instance, in pears subjected to extended cold storage, core browning has been associated with a gradual increase in LOX activity (Zhou et al., 2020), while in apples, LOX has been recognized as a major factor in core browning during cold storage (Feys et al., 1980). These findings suggest that long-term cold storage stress induces LOX activity and ROS accumulation, which, when exceeding the tissue antioxidant capacity, disrupt ROS homeostasis, accelerate membrane lipid peroxidation, and compromise cellular compartmentalization.

Apples are rich in phenolic compounds, which not only serve as substrates for PPO/POD, participating in the development of flesh browning, but also act as the principal elements accounting for the overall antioxidant capacity in fruit and vegetables, effectively, mitigating oxidative stress caused by endogenous ROS within tissues (Ma et al., 2011; Bisrat et al., 2023). Within the present study, clear differences in TF content and TAC were identified among the three sample groups (**Figures 3C**, **4B**). Both exhibited the lowest levels in the FB tissues, and a strong positive association was detected (*r* = 0.671; *p* < 0.05) (**Figure 9**). TP content showed no marked variation between FN and FB tissues (**Figure 4A**), but it is noteworthy that PAL and 4CL activities reached their highest levels in FB tissues (**Figure 5**). In the pathway of flavonoid biosynthesis, *CHI* and *F3H* were significantly up-regulated in the FB tissues, whereas *CHS* and *FLS* showed the opposite trend. Overall, the phenylpropanoid pathway was up-regulated in FB tissues, although this was not reflected in TP and TF contents (**Figure 4**). The phenylpropanoid pathway functions as a precursor process for the synthesis of flavonoids and lignin, and its activation promotes the buildup of secondary metabolites, including phenolic acids, flavonoids, and lignin, within plant tissues (Zhao et al., 2022; Chen et al., 2021; Jiang et al., 2025). The latter is closely involved in modulating postharvest quality decline-including browning, softening, and senescence—in fruits like jujube (Sang et al., 2023; Jia et al., 2023), kiwifruit (Niu et al., 2023), and peach (Liu et al., 2024) during extended cold storage, through mechanisms such as boosting tissue TAC (Zhao et al., 2022; Niu et al., 2023), reinforcing cell wall integrity (Huang et al., 2025), and postponing ripening and aging processes (Jia et al., 2023). Therefore, we speculate that activation of the phenylpropanoid pathway within FB tissues partly elevates phenolic acid levels and may facilitate lignin accumulation, thereby enhance antioxidant capacity and reinforce cell wall strength, which ultimately improves the fruit’s tolerance to prolonged cold storage.

In addition to polyphenols, antioxidants such as AsA and GSH together with enzymes like CAT, SOD, and APX form essential elements of the defense system against oxidative stress in fruit and vegetables. By engaging in ROS metabolism, they influence flesh browning processes across different fruit species (Lee et al., 2019; Zhang et al., 2025). For example, the study by Cao et al. (2011) demonstrated that higher ASA and GSH contents, along with elevated APX, SOD, and CAT activities, were beneficial for alleviating oxidative stress and low-temperature stress in fruits. Conversely, lower levels of AsA and GSH were associated with higher MDA levels, and the reduction in AsA and GSH led to ROS accumulation, ultimately resulting in increased flesh browning in apple fruit (Lee et al., 2019). In this study, AsA, GSH, CAT, and APX were identified as major contributors to tissue TAC, showing strong correlations of 0.921 (*p* < 0.001), 0.904 (*p* < 0.01), 0.874 (*p* < 0.001), and 0.928 (*p* < 0.001), respectively (**Figure 9**). Moreover, TAC exhibited a strong correlation with a* values, ROS levels, EC and MDA content (**Figure 9**). Therefore, we propose that the reduced contents of AsA and GSH, along with decreased activities of CAT and APX in FB tissues, compromised the TAC, leading to exacerbated ROS accumulation and enhanced lipid peroxidation of membranes, thereby elevating the risk of flesh browning.

## 5 Conclusion

**Figure 10:**
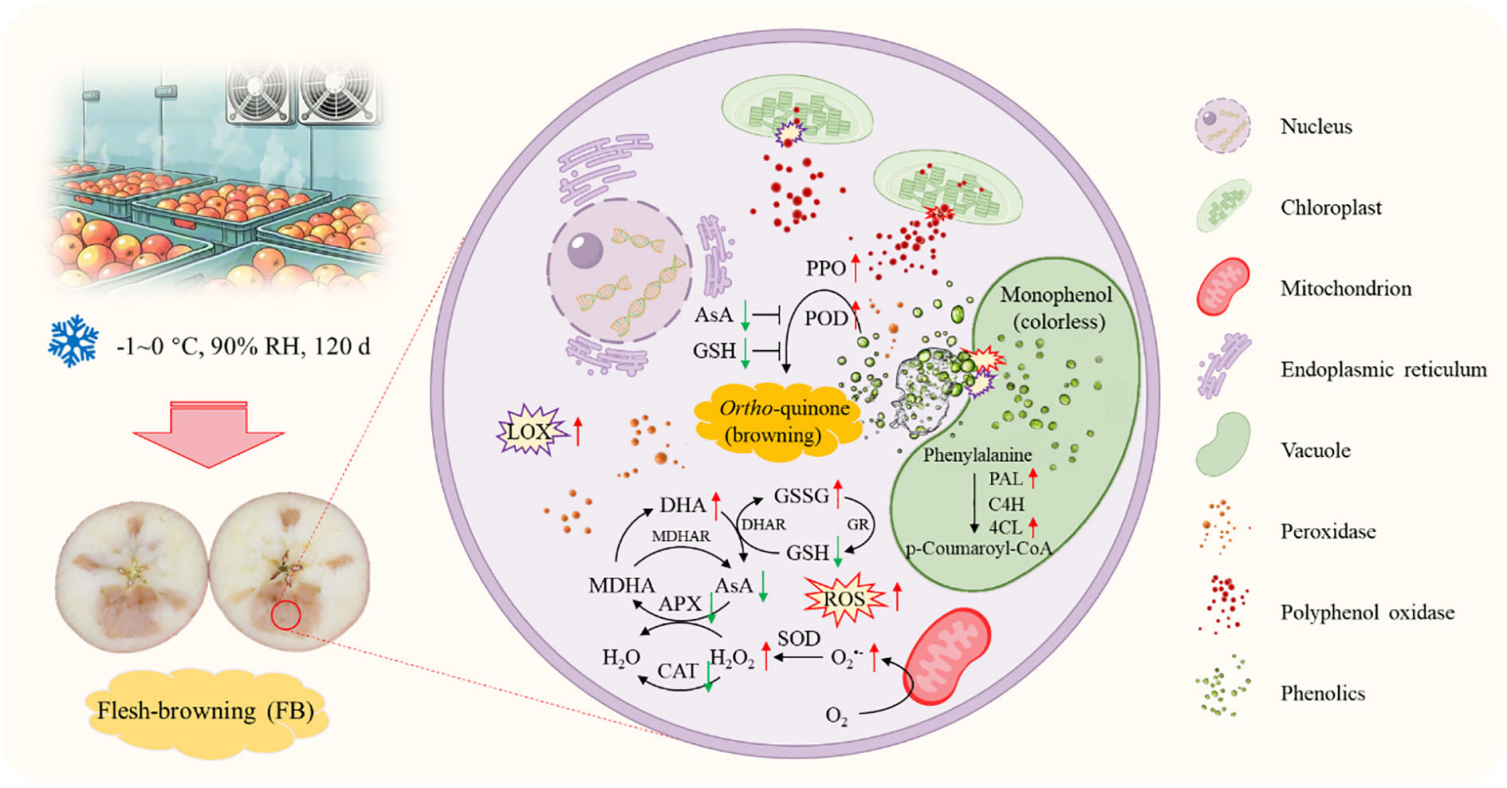
Summary diagram of flesh browning in ‘Fuji’ apples during cold storage.

In conclusion, our results demonstrated that long-term cold storage induced LOX activity and ROS accumulation, leading to the disruption of cellular membrane compartmentalization. This disruption facilitates the interaction between PPO/POD and phenolic compounds, triggering enzymatic browning. The up-regulation of the phenylpropanoid pathway partially mitigated browning by increasing phenolic acid content and enhancing cell wall strength, but it could not fully compensate for the decline in antioxidant capacity. Meanwhile, the concurrent deficiencies of non-enzymatic antioxidants (AsA/GSH) and antioxidant enzymes (CAT/APX) exacerbated ROS metabolic imbalance and membrane damage, creating a vicious cycle (**Figure 10**).

## Data Availability

The original contributions presented in the study are included in the article/**Supplementary Material**. Further inquiries can be directed to the corresponding authors.

## References

[B1] Ali H. M. El-Gizawy A. M. El-Bassiouny R. E. I. Saleh M. A. (2015). Browning inhibition mechanisms by cysteine, ascorbic acid and citric acid, and identifying PPO-catechol-cysteine reaction products. J. Food Sci. Tech 52, 3651–3659. doi: 10.1007/s13197-014-1437-0, PMID: 26028748 PMC4444905

[B2] Ali S. Rehman R. N. U. Khan A. S. Nawaz A. Naz S. Khaliq G. . (2025). Combined sodium alginate and glutathione treatment delays water chestnut (Trapa natans L.) browning by regulating oxidative stress and ascorbate glutathione cycle. Int. J. Biol. Macromol 319, 145419. doi: 10.1016/j.ijbiomac.2025.145419, PMID: 40545097

[B3] Badrunnesa A. Zhan W. D. Duan W. Y. Meng J. R. Li A. Yao Z. Y. . (2025). Transcriptome analysis reveals the mechanism of peach fruit browning during cold storage. N. Z. J. Crop Hortic. Sci. 53, 2696–2715. doi: 10.1080/01140671.2025.2501283

[B4] Bisrat M. H. Chandravanshi B. S. Redi M. Yaya E. (2023). Determination of total phenolic, total flavonoid, ascorbic acid contents and antioxidant activity of pumpkin flesh, peel and seeds. Bull. Chem. Soc Ethiop 37, 1093–1108. doi: 10.4314/bcse.v37i5.3

[B5] Cao J. K. Jiang W. B. Zhao Y. M. (2007). Instructions for postharvest physiological and biochemical experiments on fruits and vegetables (Beijing: China Light Industry Press).

[B6] Cao S. F. Yang Z. F. Cai Y. T. Zheng Y. H. (2011). Fatty acid composition and antioxidant system in relation to susceptibility of loquat fruit to chilling injury. Food Chem. 127, 1777–1783. doi: 10.1016/j.foodchem.2011.02.059

[B7] Chen O. Deng L. L. Ruan C. Q. Yi L. H. Zeng K. F. (2021). Pichia galeiformis induces resistance in postharvest citrus by activating the phenylpropanoid biosynthesis pathway. J. Agric. Food. Sci. 69, 2619–2631. doi: 10.1021/acs.jafc.0c06283, PMID: 33594880

[B8] Cheng G. W. Breen P. J. (1991). Activity of phenylalanine ammonia-lyase (pal) and concentrations of anthocyanins and phenolics in developing strawberry fruit. J. Am. Soc Hortic. Sci. 116, 986–869. doi: 10.21273/JASHS.116.5.865

[B9] Cosme P. Rodriguez A. B. Espino J. Garrido M. (2020). Plant phenolics: bioavailability as a key determinant of their potential health-promoting applications. Antioxidants 9, 1263. doi: 10.3390/antiox9121263, PMID: 33322700 PMC7764680

[B10] Dokhanieh A. Y. Aghdam M. S. (2016). Postharvest browning alleviation of Agaricus bisporus using salicylic acid treatment. Sci. Hortic. 207, 146–151. doi: 10.1016/j.scienta.2016.05.025

[B11] Feys M. Naesens W. Tobback P. Maes E. (1980). Lipoxygenase activity in apples in relation to storage and physiological disorders. Phytochemistry 19, 1009–1011. doi: 10.1016/0031-9422(80)83048-2

[B12] Fu X. D. Li X. A. Ali M. Zhao X. M. Min D. D. Liu J. . (2023). Methionine sulfoxide reductase B5 plays vital roles in tomato fruit defense response against Botrytis cinerea induced by methyl jasmonate. Postharvest Biol. Technol. 192, 112165. doi: 10.1016/j.postharvbio.2022.112165

[B13] Gapper N. E. Bowen J. K. Brummell D. A. (2023). Biotechnological approaches for predicting and controlling apple storage disorders. Curr. Opin. Biotechnol. 79, 102851. doi: 10.1016/j.copbio.2022.102851, PMID: 36446143

[B14] Glagoleva A. Y. Shoeva O. Y. Khlestkina E. K. (2020). Melanin pigment in plants: current knowledge and future perspectives. Front. Plant Sci. 11. doi: 10.3389/fpls.2020.00770, PMID: 32655591 PMC7324791

[B15] He M. Y. Ge Z. X. Hong M. Qu H. X. Duan X. W. Yun Z. . (2020). Alleviation of pericarp browning in harvested litchi fruit by synephrine hydrochloride in relation to membrane lipids metabolism. Postharvest Biol. Technol. 166, 111223. doi: 10.1016/j.postharvbio.2020.111223

[B16] Hu S. S. Wang T. L. Shao Z. Y. Meng F. L. Chen H. Wang Q. M. . (2022). Brassinosteroid biosynthetic gene SlCYP90B3 alleviates chilling injury of tomato (Solanum lycopersicum) fruits during cold storage. Antioxidants 11, 115. doi: 10.3390/antiox11010115, PMID: 35052619 PMC8773034

[B17] Huang L. J. Tao S. K. Pan Y. G. Han Z. H. Chen Y. Z. Tan Y. X. . (2025). Molecular mechanisms of low temperature-induced aberrant chilling injury in papaya fruit: Physiological and transcriptomic analysis on cell wall metabolism. Sci. Hortic. 344, 114107. doi: 10.1016/j.scienta.2025.114107

[B18] Jia L. L. Liu G. S. He J. G. (2023). UV-C delays senescence in ‘Lingwu long’ jujube fruit by regulating ROS and phenylpropanoid metabolism. Plant Physiol. Biochem. 194, 383–393. doi: 10.1016/j.plaphy.2022.11.030, PMID: 36473328

[B19] Jiang R. Wang Z. Q. Jia Y. X. Zong Y. Yang C. . (2025). Exogenous melatonin enhancing the accumulation of flavonoids and carotenoids in wolfberry fruit at cold storage. Food Res. Int. 209, 116320. doi: 10.1016/j.foodres.2025.116320, PMID: 40253210

[B20] Kampfenkel K. Vanmontagu M. Inze D. (1995). Extraction and determination of ascorbate and dehydroascorbate from plant tissue. Anal. Biochem. 225, 165–167. doi: 10.1006/abio.1995.1127, PMID: 7778771

[B21] Knobloch K. H. Hahlbrock K. (1975). Isoenzyme of p-coumarate: CoA ligase from cell suspension cultures of Glycine max. Eur. J. Biochem. 52, 311–320. doi: 10.1111/j.1432-1033.1975.tb03999.x, PMID: 240682

[B22] Kuddus M. (2018). “ Introduction to the electronic age,” in Enzymes in food technology. Eds. Singh B. Suri A. Shevkani K. ( Springer Nature, Singapore), 63–78. doi: 10.1007/978-981-13-1933-4

[B23] Lee J. Cheng L. L. Rudell D. R. Nock J. F. Watkins C. B. (2019). Antioxidant metabolism in stem and calyx end tissues in relation to flesh browning development during storage of 1-methylcyclopropene treated ‘Empire’ apples. Postharvest Biol. Technol 149, 66–73. doi: 10.1016/j.postharvbio.2018.11.015

[B24] Li B. R. Li M. Q. Liu J. Sun W. W. Min D. D. Li F. J. . (2023). Methyl salicylate pretreatment maintains quality and antioxidant capacity of fresh-cut pitaya fruit by modulating phenylpropanoid metabolism and antioxidant system. Sci. Hortic. 309, 111705. doi: 10.1016/j.scienta.2022.111705

[B25] Li J. Yao T. Xu Y. C. Cai Q. W. Wang Y. S. (2022a). Elevated CO2 exposure induces core browning in Yali pears by inhibiting the electron transport chain. Food Chem. 378, 132101. doi: 10.1016/j.foodchem.2022.132101, PMID: 35042112

[B26] Li F. J. Zhang X. H. Song B. C. Li J. Z. Shang Z. L. Guan J. F. (2013). Combined effects of 1-MCP and MAP on the fruit quality of pear (Pyrus bretschneideri Reld cv. Laiyang) during cold storage. Sci. Hortic. 164, 544–551. doi: 10.1016/j.scienta.2013.10.018

[B27] Li X. A. Li M. L. Ji N. N. Jin P. Zhang J. H. Zheng Y. H. . (2019). Cold plasma treatment induces phenolic accumulation and enhances antioxidant activity in fresh-cut pitaya (Hylocereus undatus) fruit. LWT 115, 108447. doi: 10.1016/j.lwt.2019.108447

[B28] Li Z. L. Li B. R. Li M. Q. Fu X. D. Zhao X. M. Min D. D. . (2022b). Hot air pretreatment alleviates browning of fresh-cut pitaya fruit by regulating phenylpropanoid pathway and ascorbate-glutathione cycle. Postharvest Biol. Technol. 190, 111954. doi: 10.1016/j.postharvbio.2022.111954

[B29] Liu J. Y. Lin Y. F. Lin H. T. Lin M. S. Fan Z. Q. (2021). Impacts of exogenous ROS scavenger ascorbic acid on the storability and quality attributes of fresh longan fruit. Food Chem. 12, 100167. doi: 10.1016/j.fochx.2021.100167, PMID: 34870143 PMC8626660

[B30] Liu Y. D. Wu J. L. Li Y. Deng W. Cao K. Li Z. G. . (2024). Metabolism and transcriptional regulation in chilling injury development of nectarine fruit during postharvest cold storage. Postharvest Biol. Technol. 210, 112748. doi: 10.1016/j.postharvbio.2023.112748

[B31] Luo Z. S. Wu X. Xie Y. Chen C. (2012). Alleviation of chilling injury and browning of postharvest bamboo shoot by salicylic acid treatment. Food Chem. 131, 456–461. doi: 10.1016/j.foodchem.2011.09.007

[B32] Ma X. W. Wu H. X. Liu L. Q. Yao Q. S. Wang S. B. Zhan R. L. . (2011). Polyphenolic compounds and antioxidant properties in mango fruits. Sci. Hortic. 129, 102–107. doi: 10.1016/j.scienta.2011.03.015

[B33] Mao L. C. Pang H. Q. Wang G. Z. Zhu C. G. (2007). Phospholipase D and lipoxygenase activity of cucumber fruit in response to chilling stress. Postharvest Biol. Technol. 44, 42–47. doi: 10.1016/j.postharvbio.2006.11.009

[B34] Mirshekari A. Madani B. Yahia E. M. Golding J. B. Vand S. H. (2019). Postharvest melatonin treatment reduces chilling injury in sapota fruit. J. Sci. Food Agric. 100, 1897–1903. doi: 10.1002/jsfa.10198, PMID: 31825530

[B35] Morariu P. A. Sestras A. F. Andrecan A. F. Borsai O. Bunea C. L. Militaru M. . (2025). Apple cultivar responses to fungal diseases and insect pests under variable orchard conditions: a multisite study. Crops 5, 30. doi: 10.3390/crops5030030

[B36] Niu Y. X. Ye L. X. Wang Y. Shi Y. B. Liu Y. J. Luo A. W. (2023). Transcriptome analysis reveals salicylic acid treatment mitigates chilling injury in kiwifruit by enhancing phenolic synthesis and regulating phytohormone signaling pathways. Postharvest Biol. Technol. 205, 112483. doi: 10.1016/j.postharvbio.2023.112483

[B37] Sang Y. Y. Yang W. T. Zhang W. D. Guo M. R. Cheng S. B. Yu X. H. . (2023). Abscisic acid enhances storability of winter jujube by regulating cell wall and phenylpropane metabolisms during cold storage. J. Agric. Food Res. 14, 100859. doi: 10.1016/j.jafr.2023.100859

[B38] Sethi S. Joshi A. Arora B. Bhowmik A. Sharma R. R. Kumar P. (2020). Significance of FRAP, DPPH, and CUPRAC assays for antioxidant activity determination in apple fruit extracts. Eur. Food Res. Technol. 246, 591–598. doi: 10.1007/s00217-020-03432-z

[B39] Sidhu R. S. Bound S. A. Swarts N. D. (2023). Internal flesh browning in apple and its predisposing factors—a review. Physiologia 3, 145–172. doi: 10.3390/physiologia3020012

[B40] Sogvar O. B. Razavi F. Babiei V. Gohari G. (2020). Postharvest application of L-cysteine to prevent enzymatic browning of “Stanley’’ plum fruit during cold storage. J. Food Process. Preserv 44, e14788. doi: 10.1111/jfpp.14788

[B41] Sultan M. Z. Farouk K. A. Elbagoury M. M. Yahia E. M. (2025). Trends in biochemical, anatomical mechanisms and molecular aspects in enzymatic browning of apples: a review. Eur. Food Res. Technol. 251, 3305–3326. doi: 10.1007/s00217-025-04824-9

[B42] Tang T. T. Xie X. F. Ren X. Wang W. J. Tang X. M. Zhang J. . (2020). A difference of enzymatic browning unrelated to PPO from physiology, targeted metabolomics and gene expression analysis in Fuji apples. Postharvest Biol. Technol. 170, 111323. doi: 10.1016/j.postharvbio.2020.111323

[B43] Vámos-Vigyázó L. Haard N. F. (1981). Polyphenol oxidases and peroxidases in fruits and Vegetables. Food Sci. Technol. 15, 49–127. doi: 10.1080/10408398109527312, PMID: 6794984

[B44] Wang J. H. Li F. J. Sun W. W. Ali M. Li B. R. Zhang X. Y. . (2024). Role of sugar and energy metabolism in apple flesh browning during cold storage. Sci. Hortic. 326, 112758. doi: 10.1016/j.scienta.2023.112758

[B45] Wang J. H. Li F. J. Zhang X. Y. Sun W. W. Ali M. Li X. A. . (2023). Combined transcriptomic and targeted metabolomic analysis reveals the mechanism of flesh browning in cold stored ‘Fuji’ apple fruit. Sci. Hortic. 320, 112195. doi: 10.1016/j.scienta.2023.112195

[B46] Wang Z. Q. Pu H. L. Shan S. S. Zhang P. Li J. K. Song H. M. . (2021). Melatonin enhanced chilling tolerance and alleviated peel browning of banana fruit under low temperature storage. Postharvest Biol. Technol. 179, 111571. doi: 10.1016/j.postharvbio.2021.111571

[B47] Yoruk R. Marshall M. R. (2003). Physicochemical properties and function of plant polyphenol oxidase: a review. J. Food Biochem. 27, 361–422. doi: 10.1111/j.1745-4514.2003.tb00289.x

[B48] Zhang X. H. Min D. D. Li F. J. Ji N. N. Meng D. M. Li L. (2017). Synergistic effects of l-arginine and methyl salicylate on alleviating postharvest disease caused by Botrysis cinerea in tomato fruit. J. Agric. Food Chem. 65, 4890–4896. doi: 10.1021/acs.jafc.7b00395, PMID: 28535671

[B49] Zhang M. Z. Zhu C. S. Na Q. T. Cao H. Tian C. Liu G. S. . (2025). The impact of fruit size on internal browning in pineapples. J. Food Sci. 90, e17622. doi: 10.1111/1750-3841.17622, PMID: 39898989

[B50] Zhao J. Ao M. He X. Li W. Deng L. Zeng K. . (2022). Changes in phenolic content, composition, and antioxidant activity of blood oranges during cold and on-tree storage. J. Integr. Agric. 21, 3669–3683. doi: 10.1016/j.jia.2022.09.011

[B51] Zhou H. S. Tian M. Y. Huang W. Luo S. F. Hu H. L. Zhang Y. T. . (2020). Physiological and transcriptomic analysis of ‘Whangkeumbae’ pear core browning during low-temperature storage. Gene Expr. Patterns 36, 119113. doi: 10.1016/j.gep.2020.119113, PMID: 32325218

[B52] Zhu L. J. Hu W. F. Murtaza A. Iqbal A. Li J. X. Zhang J. . (2022). Eugenol treatment delays the flesh browning of fresh-cut water chestnut (Eleocharis tuberosa) through regulating the metabolisms of phenolics and reactive oxygen species. Food Chem. 14, 100307. doi: 10.1016/j.fochx.2022.100307, PMID: 35492256 PMC9043673

