# Supplementary material for: ROS-mediated membrane damage and antioxidant imbalance drive apple flesh browning during cold storage
Source: Front Plant Sci. 2025 Dec 1;16:1718635. doi: 10.3389/fpls.2025.1718635 (PMC12703709; doi:10.3389/fpls.2025.1718635)
Supplement: Supplementary file 1 [file Table1.docx]

**SUPPLEMENTARY FILE**

**ROS-mediated membrane damage and antioxidant imbalance**

**drive apple flesh browning during cold storage**

Jihan Wang^1†^, Fujun Li^1†^, Bingru Li^1^, Ling Li^2^, Jing Shang^1^, Xiaodong Fu^1^, Xiuming Zhao^1^, Xiaoan Li^1^, Xinhua Zhang^1*^, Zienab F. R. Ahmed^3*^

^1^College of Agricultural Engineering and Food Science, Shandong University of Technology, Zibo, 255049, Shandong, PR China

^2^College of Food and Biological Engineering, Beijing Vocational College of Agriculture, Fangshan District, Beijing 102442, PR China

^3^Integrative Agriculture Department, College of Agriculture and Veterinary Medicine, United Arab Emirates University, Al Ain 15551, UAE

*** Correspondence:**

Xinhua Zhang
zxh@sdut.edu.cn

Zienab F.R. Ahmed
Zienab.ahmed@uaeu.ac.ae

**^†^Contributed equally to this work**

**Abbreviations**

| Abbreviation | Full name |
| --- | --- |
| 4CL | 4-coumarate: CoA ligase |
| APX | Ascorbic acid peroxidase |
| AsA | Ascorbic acid |
| C4H | Cinnamate 4-hydroxylase |
| CAT | Catalase |
| DHA | Dehydroascorbate |
| EC | Energy charge |
| FB | Flesh-browning |
| FN | Flesh-normal |
| FRAP | Ferric reducing antioxidant power |
| GSH | Reduced glutathione |
| GSSG | Glutathione disulfide |
| LOX | Lipoxygenase |
| MDA | Malondialdehyde |
| NBT | Nitro blue tetrazolium |
| PAL | Phenylalanine ammonia-lyase |
| POD | Peroxidase |
| PPO | Polyphenol oxidase |
| PVPP | Polyvinylpolypyrrolidone |
| ROS | Reactive oxygen species |
| SOD | Superoxide dismutase |
| SSC | Soluble solids content |
| TAC | Total antioxidant capacity |
| TA | Titratable acid |
| TF | Total flavonoids |
| TP | Total polyphenolics |
| U | Unit |
